# Identification of a Novel Gene Encoding the Specialized Alanine Decarboxylase in Tea (*Camellia sinensis*) Plants

**DOI:** 10.3390/molecules24030540

**Published:** 2019-02-01

**Authors:** Peixian Bai, Kang Wei, Liyuan Wang, Fen Zhang, Li Ruan, Hailin Li, Liyun Wu, Hao Cheng

**Affiliations:** 1 National Center for Tea Improvement, Tea Research Institute Chinese Academy of Agricultural Sciences, Hangzhou 310008, China; baipeixian2018@outlook.com (P.B.); weikang@tricaas.com (K.W.); zhangfen2008.cool@163.com (F.Z.); ruanli@tricaas.com (L.R.); lihailin@tricaas.com (H.L.); wuly@tricaas.com (L.W.); 2 College of Horticulture and Forestry Science, Huazhong Agricultural University, Wuhan 430070, China

**Keywords:** theanine, ethylamine, alanine decarboxylase, *Camellia sinensis*, enzymatic activity, gene expression, nitrogen metabolism

## Abstract

Theanine, a unique amino acid in *Camellia sinensis*, accounts for more than 50% of total free amino acids in tea and has a significant contribution to the quality of green tea. Previous research indicated that theanine is synthesized from glutamic acid (Glu) and ethylamine mainly in roots, and that theanine accumulation depends on the availability of ethylamine which is derived from alanine (Ala) decarboxylation catalyzed by alanine decarboxylase (AlaDC). However, the specific gene encoding AlaDC protein remains to be discovered in tea plants or in other species. To explore the gene of AlaDC in tea plants, the differences in theanine contents and gene expressions between pretreatment and posttreatment of long-time nitrogen starvation were analyzed in young roots of two tea cultivars. A novel gene annotated as *serine decarboxylase* (*SDC*) was noted for its expression levels, which showed high consistency with theanine content, and the expression was remarkably high in young roots under sufficient nitrogen condition. To verify its function, full-length complementary DNA (cDNA) of this candidate gene was cloned from young roots of tea seedlings, and the target protein was expressed and purified from *Escherichia coli* (*E. coli*). The enzymatic activity of the protein for Ala and Ser was measured in vitro using ultra-performance liquid chromatography coupled with mass spectrometry (UPLC-MS). The results illustrated that the target protein could catalyze the decarboxylation of Ala despite of its high similarity with SDC from other species. Therefore, this novel gene was identified as *AlaDC* and named *CsAlaDC*. Furthermore, the gene expression levels of *CsAlaDC* in different tissues of tea plants were also quantified with quantitative real-time PCR (qRT-PCR). The results suggest that transcription levels of *CsAlaDC* in root tissues are significantly higher than those in leaf tissues. That may explain why theanine biosynthesis preferentially occurs in the roots of tea plants. The expression of the gene was upregulated when nitrogen was present, suggesting that theanine biosynthesis is regulated by nitrogen supply and closely related to nitrogen metabolism for *C. sinensis*. The results of this study are significant supplements to the theanine biosynthetic pathway and provide evidence for the differential accumulation of theanine between *C. sinensis* and other species.

## 1. Introduction

Tea is the most widely consumed non-alcoholic beverage in the world [1,2]. An important reason for its popularity is the unique taste and flavor of tea, which is provided by secondary metabolites unique to tea plants (*Camellia sinensis*) [3,4,5]. Theanine (γ-glutamylethylamine) is one such unique metabolite, which contributes to the umami flavor of green tea [6,7]. Theanine, a unique amino acid of *C. sinensis*, accounts for more than 50% of total amino acids in tea plants [8]. Aside from its special taste, theanine also has many health promotion effects on humans [9,10,11,12,13,14,15,16], such as promoting concentration and relaxation, protecting against vascular diseases, and counteracting the effects of caffeine. Moreover, theanine performs crucial functions in the physiological processes occurring in tea plants. Due to its high content (1–2% dry weight (DW)) in tea [17,18,19,20,21] and the two amines it contains within a single molecule [6], theanine is considered as a nitrogen pool inside the plant [22] and it is involved in nitrogen metabolism in *C. sinensis*.

**Figure 1:**
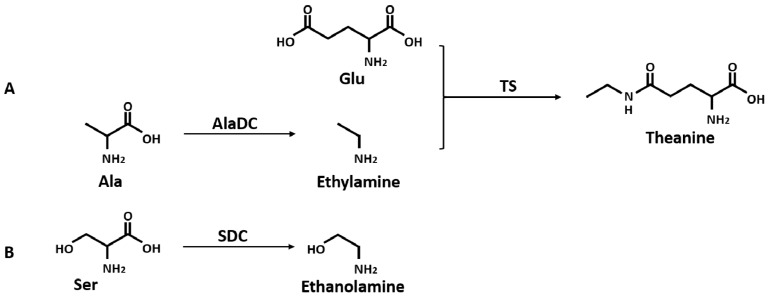
Biosynthetic pathway of theanine (**A**), and decarboxylation of Ser (**B**). AlaDC, alanine decarboxylase; TS, theanine synthetase; SDC, serine decarboxylase.

Theanine is synthesized from glutamic acid (Glu) and ethylamine mainly in roots [23,24,25,26] (Figure 1A) and transported to leaves via the xylem [27,28]. In its aboveground parts, theanine is hydrolyzed back to Glu and ethylamine [29], and then it is involved in the following nitrogen cycle [30]. In *C. sinensis*, the conversion of Glu and ethylamine into theanine is catalyzed by theanine synthetase (TS, EC 6.3.1.6) which has dramatically high homology to glutamine synthetase (GS, EC 6.3.1.2) [31]. A precursor of theanine, Glu, is produced from the GS/GOGAT (glutamine-2-oxoglutarate aminotransferase, EC 1.4.1.14) cycle and glutamate dehydrogenase (GDH, EC 1.4.1.2) pathway [32,33]. Another precursor of theanine, ethylamine, is derived from alanine (Ala) decarboxylation catalyzed by AlaDC [26]. Previous studies proposed that alanine and acetaldehyde might be the precursors of ethylamine in plant tissues [34,35]. Takeo’s study [26] revealed that the incorporation of ^14^C from [U-^14^C]-alanine into theanine was faster than that from [1-2^14^C]-acetaldehyde, resulting in a conclusion that alanine was closer to theanine in the biosynthetic pathway than acetaldehyde. The conversion of radioactive [U-^14^C]-alanine into theanine was also confirmed by Deng et al. [23]. A recent study reported that the availability of ethylamine was the restricting factor for the differential accumulation of theanine between *C. sinsnsis* and other species [36]. Therefore, it could be concluded that CsAlaDC plays a vital role in the occurrence of theanine in tea plants by producing ethylamine from Ala [26,36,37]. Although several studies reported that the activity of AlaDC was observed from the homogenate of tea seedlings, there were few studies regarding the specialized enzyme catalyzing the formation of ethylamine [38]. The specific gene coding the AlaDC protein remains to be reported in tea plants or any other species.

In this study, a nitrogen starvation treatment was performed on tea plants of two cultivars “Zhongcha 108” (ZC108) and “Zhongcha 302” (ZC302). By comparing the amino acid contents and gene expression profiles in the roots of tea plants under nitrogen starvation treatment to those of the control group, we identified a candidate gene of the AlaDC (annotated as serine decarboxylase). Then, the target gene was cloned from the root tissues of tea plants. After expression and purification from *E. coli*, the enzymatic activity of the target protein was measured in vitro to investigate the bioactivity of the candidate gene. The present study might provide important genetic information for the theanine biosynthetic pathway and a better understanding of theanine accumulation preference in tea plants rather than in other species.

## 2. Results and Discussion

### 2.1. Effects of Nitrogen Starvation on Content of Free Amino Acids in Tea Roots

**Figure 2:**
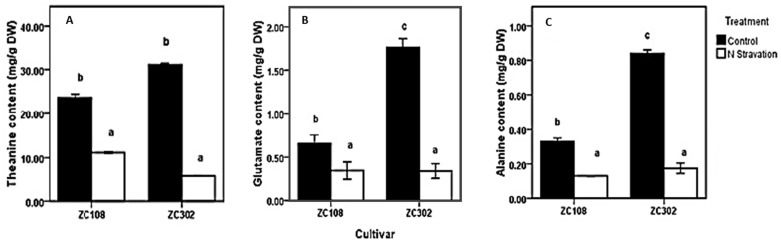
Effects of nitrogen starvation on content of amino acids involved in theanine biosynthetic pathway (**A**–**C**) in the root of tea plants. ZC108 refers to tea cultivar “Zhongcha 108”, and ZC302 refers to tea cultivar “Zhongcha 302”. Error bars represent the standard errors across three biological replicates. Different letters above the bars indicate significant differences at *p* < 0.05.

To investigate the effects of nitrogen availability on nitrogen metabolism in the roots of tea plants, a nitrogen starvation treatment was conducted on tea seedlings. The contents of theanine and two other amino acids, Glu and Ala, were measured. Glu and Ala were reported to be directly involved in theanine biosynthesis in the roots of tea plants. As shown in Figure 2, after the treatment with nitrogen starvation, the content of amino acids in roots decreased significantly. The content of theanine (11.77 mg/g DW) in ZC108 reduced by more than half, compared to that in the control group (23.64 mg/g DW). In ZC302, theanine content decreased to less than one-fifth when suffering from nitrogen starvation. Amino acids in plants play a key role in many physiological processes, especially in nitrogen assimilation and metabolism. Theanine is the predominant amino acid in every organ of the tea plants and accounts for more than 50% of the total free amino acids [17,18,19,20,21]. A single molecule of theanine contains two amines, just like glutamine [6]. Therefore, theanine is considered as the nitrogen pool of the tea plants [22] and is involved in nitrogen metabolism. The results obtained above indicate a correlation between the nitrogen availability and amino-acid accumulation in tea plants. Consistently, when plants were subjected to a limited nitrogen condition, the content of free amino acids in plants declined significantly [39,40,41], suggesting that the initial nitrogen required by the whole plant might be supplied by ammonium released from free amino acids [39]. The content of theanine, as well as of total free amino acids, in root tissues decreased notably when tea plants were treated with nitrogen starvation [42], and the content was significantly raised by increasing nitrogen supply [43], suggesting that biosynthesis of theanine in tea plants relies heavily on nitrogen supply.

### 2.2. Identification of a Candidate Gene of *CsAlaDC*

**Figure 3:**
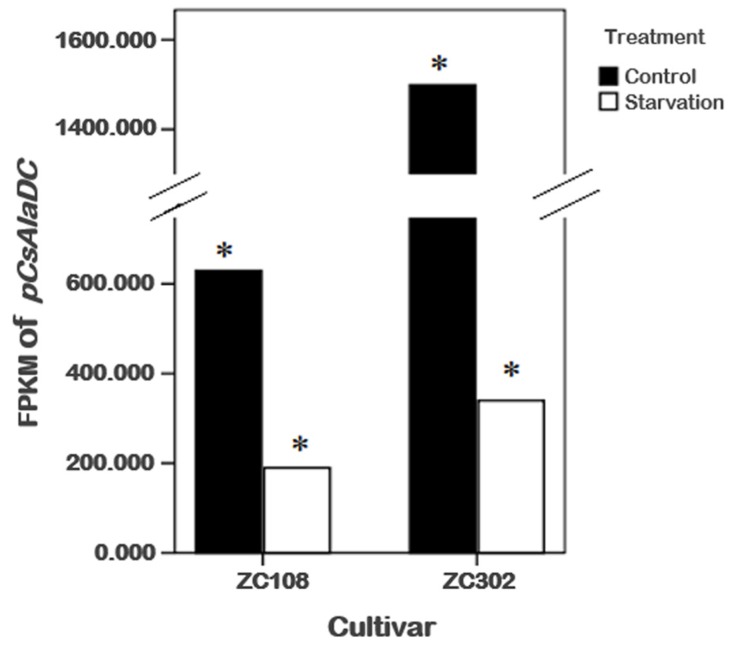
Effects of nitrogen starvation on gene expression levels of *pCsAlaDC* in the roots of tea plants. * represents a significant difference at *p* < 0.05.

To obtain genes involved in nitrogen starvation, the roots of two tea cultivars representing two genotypes with or without nitrogen treatment were used for RNA sequencing (RNA-Seq). After screening, assembling, and hierarchical clustering, a total of 341,035 unigenes were detected from four samples. By running the Tigr Gene Indices Clustering Tools (TGICL) program, we obtained 100,626 non-redundant sequences, including 28,645 clusters and 71,981 singletons. As shown in Table 1, a total of 91,650 and 98,406 unigenes were obtained from ZC302 and ZC108, respectively. Based on differential expression analysis, 7985 genes were identified from ZC302, and 17,824 genes were identified from ZC108, and their transcription levels were observed to be significantly changed when treated with nitrogen starvation. Among the differentially expressed genes (DEGs), 742 genes were found to be upregulated and 128 genes were found to be downregulated in both ZC302 and ZC108. The shared genes were annotated by aligning the putative protein sequences against the protein database of Non-redundant protein sequence (NR) and Swiss-Prot. A candidate gene, annotated as a *serine decarboxylase* (*SDC*), aroused our attention because its expression levels were remarkably high under sufficient nitrogen condition, and dramatically decreased when subjected to nitrogen starvation in both cultivars (shown in Figure 3). This result indicated that the gene was significantly regulated by nitrogen availability. The results mentioned above suggested that theanine and Ala contents in tea roots were also enormously regulated by nitrogen supply. An important enzyme for theanine biosynthesis in *C. sinensis* is AlaDC, the gene of which remains to be found. Since the chemical structure of Ala is very similar to that of Ser (Figure 1B), it could be hypothesized that the candidate gene might have the activity to catalyze the decarboxylation of Ala. We putatively named the candidate gene *CsAlaDC* (*pCsAlaDC*).

### 2.3. Cloning of pCsAlaDC from Camellia sinensis

**Figure 4:**
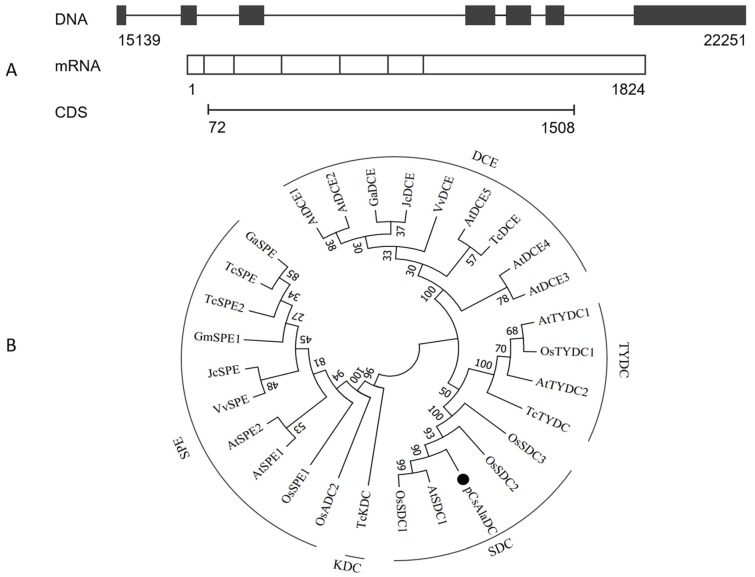
Cloning and sequence analysis of *pCsAlaDC* from tea plants. (**A**) Prediction of the gene structure using online software Splign. The genome sequence of the gene was obtained from the “Shuchazao” genome. (**B**) Phylogenetic relationship of pCsAlaDC from the tea plants with amino-acid decarboxylases from other species. The sequences of amino-acid decarboxylases from seven plant species were obtained from the UniProt database. The alignment was conducted using the ClustalW method, and the phylogenetic analysis was performed using the neighbor-joining method with MEGA7 software; pCsAlaDC is denoted by a dot. SDC, serine decarboxylase; TYDC, tyrosine decarboxylase; DCE, glutamate decarboxylase; KDC, lysine decarboxylase; SPE or ADC, arginine decarboxylase. At, *Arabidopsis thaliana*; Os, *Oryza sativa*; Gm, *Glycine max*; Tc, *Theobroma cacao*; Ga, *Gossypium arboreum*; Vv, *Vitis vinifera*; Jc, *Jatropha curcas*.

To determine the biological function of *pCsAlaDC*, the candidate gene was cloned from the roots of “LongJing 43” (LJ43). The complementary DNA (cDNA) sequence of *pCsAlaDC* was aligned against genomic sequences of “Shuchazao”. The results showed that the *pCsAlaDC* gene consisted of seven exons (as shown in Figure 4A) with an average length of 260 bp. The candidate gene was predicted to encode a protein consisting of 478 amino-acid residues with a calculated molecular mass of 53.6 kDa and a theoretical isoelectric point of 5.83.

In order to make further predictions of *pCsAlaDC* function, a BLASTP homology search of the NCBI database was conducted. Based on this search, we constructed a phylogenetic tree. The results showed that the deduced protein sequence of the *pCsAlaDC* gene shared high similarity with SDCs from other plant species (Figure S1, Supplementary Materials), such as AtSDC1 (74.3% identity) and OsSDC1 (78.7% identity). Phylogenetic analysis revealed that the decarboxylases from different species clustered together based on the differences in substrates they catalyzed. As shown in Figure 4B, SDCs, tyrosine decarboxylases (TYDCs), glutamate decarboxylases (DCEs), a lysine decarboxylase (KDC), and arginine decarboxylases (SPEs or ADCs) were grouped into five clades, and the deduced *pCsAlaDC* protein was clustered with SDC proteins, indicating that the *pCsAlaDC* protein might function as an SDC. A recent study reported that plant decarboxylases shared a common evolutionary lineage, whereas their significant sequence divergence resulted in intricate evolutionary relationships between the enzymes and their functional divergence [44]. The functions of enzymes were predicted by aligning their sequence homologies against the characterized closest decarboxylases; however, this approach was not always infallible [45]. For example, *AtSDC* was initially annotated as a histidine decarboxylase (HDC) member [46]. Until recently, SDC enzymes were considered to be functionally conserved. However, new biochemical evidence suggested that these enzymes were functionally diverged in plants [47]. Bunsupa et al. reported that several amino-acid replacements in a decarboxylase could change its substrate specificity [48]. Based on these findings, it could be deduced that, although the candidate gene was annotated as an SDC, it might function as an AlaDC.2.4 target protein, which could catalyze the decarboxylation of alanine rather than serine.

**Figure 5:**
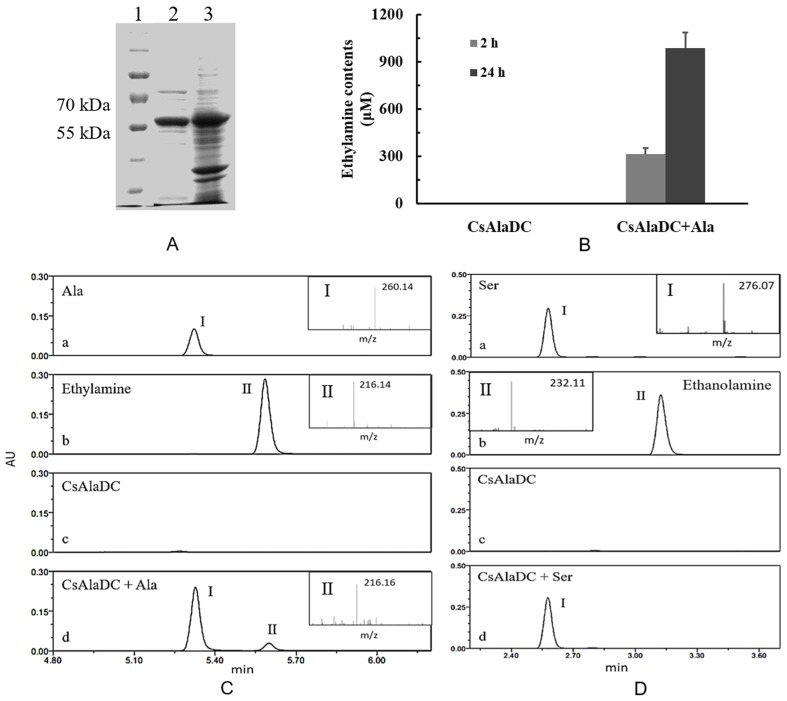
Expression, purification, and enzymatic activity of pCsAlaDC protein. (**A**) Gel image of pCsAlaDC protein expressed in *Escherichia coli*. Lanes 1–3 represent the marker, target protein, and total protein. (**B**) Measurement of AlaDC activity of the target protein. The assays were conducted by detecting the formation of ethylamine in the reaction mixture containing alanine after 2 h and 24 h of reaction at 35 °C. Error bars indicate the standard errors across three replicates. (**C**, **D**) Chromatogram for the decarboxylation of Ala (**C**) and Ser (**D**) catalyzed by pCsAlaDC. C-a~d (or D-a~d), the reaction buffer containing Ala (or Ser), ethylamine (or ethanolamine), pCsAlaDC, pCsAlaDC, and Ala. Peak I represents substrate Ala (or Ser), peak II represents the production ethylamine (or ethanolamine).

In order to determine the enzymatic activity of pCsAlaDC, the full-length cDNA of the candidate gene was cloned into prokaryotic expression vector pET28b. The recombinant construct was named *pET28b (+) pCsAlaDC*. After expression and purification, the purified protein was obtained with a molecular mass of approximately 55 kDa (shown in Figure 5A), which agreed with the predicted result (53.6 kDa).

Based on the results of online BLAST, Ser was predicted to be a possible substrate of the target protein, while, Ala, the precursor of ethylamine, is very similar to Ser in its chemical structure (shown in Figure 1). As standard amino acids, both of them contain an alpha-amine (–NH_2_) and an alpha-carboxyl (–COOH) functional group. The difference lies in that the side chain is a hydrogen (–H) for Ala, while it is a hydroxyl (–OH) for Ser. In order to determine whether pCsAlaDC could catalyze the decarboxylation of Ala or Ser, the enzymatic activity assay of the target protein was carried out. The results showed that pCsAlaDC catalyzed the decarboxylation of Ala rather than that of Ser. As shown in Figure 5, ethylamine was produced from Ala after 2 h of reaction catalyzed by pCsAlaDC (Figure 5B, C-d), and accumulated threefold after 24 h of reaction (Figure 5B). On the other hand, the product of Ser decarboxylation, ethanolamine, was not detected in the reaction mixture containing Ser (Figure 5D-d) even after 24-h incubation. These results demonstrated that pCsAlaDC could function as an alanine decarboxylase in *C. sinensis*, which confirmed our previous hypothesis. Based on these findings, the candidate gene was named *CsAlaDC*. Since CsAlaDC catalyzed the production of ethylamine, a key metabolite for theanine accumulation [36], it could be deduced that CsAlaDC plays a crucial role in the theanine biosynthetic pathway. However, little research focused on AlaDC in other species, which might be attributed to the fact that only a few species synthesize and accumulate theanine aside from *Camellia* [49], suggesting that few species have a demand for ethylamine. The narrower distribution of theanine and ethylamine across species suggests a narrow spread of *AlaDC* among species, revealing that *AlaDC* might be a new gene which fixed in the population and acquired its function very recently. A previous study demonstrated that amino-acid replacements outside the active site of an enzyme could result in a novel function, due to an alteration in substrate specificity [50]. Lysine/ornithine decarboxylase (L/ODC) is a bifunctional enzyme, and it was confirmed that amino-acid substitutions from histidine to tyrosine or to phenylalanine at position 344 of the enzyme were important for a shift from ODC to LDC activity [48]. However, it requires further effort to determine whether AlaDC in *C. sinensis* acquired its function from SDC in the same way.

Amines detected in this study were converted to compounds by reacting with AQC (6-aminoquinolyl-*N*-hydroxysuccinimidyl) reagent preceding the ultra-performance liquid chromatography coupled with mass spectrometry (UPLC-MS) analysis [51,52], which explained why the *m*/*z* ratios of amines in the MS analysis were greater than their molecular weights.

### 2.4. CsAlaDC Gene Was Expressed at a High Level in the Roots Where Theanine Was Synthesized

**Figure 6:**
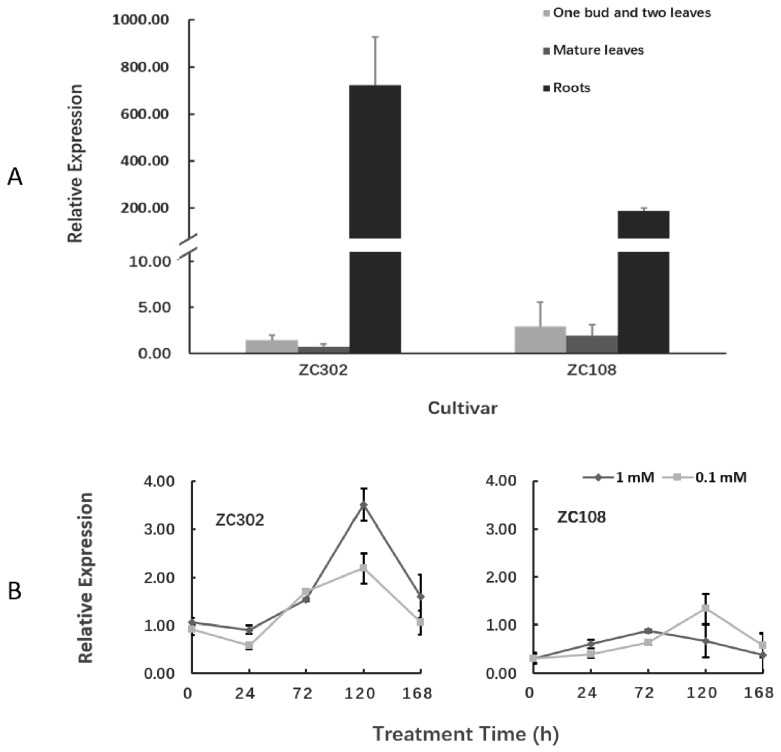
Expression of *CsAlaDC* in tea plants. (**A**) Expression of *CsAlaDC* in several tissues of tea cultivars. (**B**) Nitrogen treatment effect on the expression levels of *CsAlaDC* in the roots of tea plants. The reported concentrations (1 mM and 0.1 mM) refer to the level of NH_4_NO_3_ treatment for tea seedlings. The relative expressions of the gene were quantified using qRT-PCR and normalized to *GAPDH*. Error bars indicate the standard errors over three biological replicates.

The transcription levels of the *CsAlaDC* gene in different tissues of tea plants were detected using qRT-PCR (Figure 6A). The gene was expressed in all three tissues, but its expression was much higher in roots than in leaves. Especially in ZC302, the expression level of *CsAlaDC* in roots was 489-fold that in shoots, and 964-fold that in mature leaves. This result was consistent with the previous findings that alanine was decarboxylated at a higher rate in roots than in other organs [23,24]. Deng et al. [23] reported that the exogenous NH_3_ was converted to theanine mainly in tea roots, suggesting that theanine biosynthesis occurred mainly in roots. The precursor of ethylamine, Ala, accumulated at a higher level in roots than in other issues, and the incorporation of radioactivity from [U-^14^C]-alanine into theanine was also higher in roots than in other issues [24], indicating that the decarboxylation of alanine preferentially occurred in roots. These results provide vital evidence for the tissue preference of theanine biosynthesis in tea plants.

It is notable that the expression level of *CsAlaDC* in root tissues of ZC302 was 2–3-fold higher than that in root tissues of ZC108, which is in accordance with the results previously obtained from the transcriptome (Figure 3). Consistently, the content of theanine in ZC302 was higher than that in ZC108 (Figure 2A). The differential expressions of the gene might explain the differences in theanine accumulation between two cultivars.

The expression levels of *CsAlaDC* in roots of tea plants were also quantified to investigate the effect of nitrogen supply on the function of this gene. Tea seedlings were treated with NH_4_NO_3_ at 0.1 mM (a low concentration for tea) and 1 mM (an appropriate or normal concentration). Figure 6B shows that the expression levels of *CsAlaDC* in both cultivars did not change significantly after treatment with nitrogen for 24 h. However, after three days of treatment, the expression levels of this gene were upregulated in both ZC302 and ZC108, and reached their peaks in the next two days, before declining. That might be because, after five days of treatment, the tea plants accumulated enough CsAlaDC proteins in response to nitrogen supply. In ZC302, the normal level of nitrogen supply had a stronger upregulatory effect on the expression level of *CsAlaDC* than the low level of nitrogen supply, while this result was opposite to that in ZC108. Previous studies revealed that ZC302 had higher yield (1645 kg fresh leaf/hectare) than ZC108 (1211 kg fresh leaf/ha) in the field, which might be attributed to the fact that ZC302 has a greater growth potential than ZC108 [53]. Meanwhile, the two cultivars had different nitrogen use efficiency (NUE) since ZC302 had a higher NH_4_^+^ uptake efficiency than ZC108. The differences in growth potential and NUE between both cultivars might be the reason for the differential expressions of *CsAlaDC* in response to different levels of nitrogen supply.

## 3. Materials and Methods

### 3.1. Plant Materials and Growth Conditions

To investigate the nitrogen starvation effect on nitrogen metabolism in tea plants, two cultivars (ZC108 and ZC302) of different genotypes were used in the experiment. Tea seedlings with five or six leaves were chosen and planted in nutrient solution containing 0.1 mM NH_4_NO_3_ for eight weeks and the solution was replaced twice a week. The solution beneficial for tea plants contained the following nutrients (mmol/L): NH_4_NO_3_ (1.0), KH_2_PO_4_ (0.07), K_2_SO_4_ (0.3), MgSO_4_·7H_2_O (0.67), CaCl_2_ (0.53), and Al_2_(SO_4_)_3_·18H_2_O (0.035), (μmol/L) ethylenediaminetetraacetic acid (EDTA)·Na_2_Fe (4.2), H_3_BO_3_ (7.0), MnSO_4_·H_2_O (1.0), ZnSO_4_·7H_2_O (0.67), CuSO_4_·5H_2_O (0.13), and (NH_4_)_6_Mo_7_O_24_·4H_2_O (0.047). The pH of the nutrient solution was adjusted to 5.6 using H_2_SO_4_ and NaOH. Tea seedlings grew in controlled conditions of 16 h light/8 h dark (450 μmol·m^−2^·s^−1^ light intensity) at 20–25 °C.

To investigate the effect of nitrogen supply at different levels on theanine accumulation, tea seedlings were precultured in nutrient solution without nitrogen for two weeks and then treated with NH_4_NO_3_ at 0.1 mM (a low concentration for tea) and 1 mM (an appropriate or normal concentration). After treatment, the samples of young roots were carefully washed with distilled water, before being immediately frozen in liquid N_2_, and stored at −70 °C until extraction.

### 3.2. Determination of Free Amino-Acid Content

The harvested roots were freeze-dried and ground finely. Free amino acids in roots were extracted with H_2_O (1/100, *w*/*v*) in a water bath at 100 °C for 5 min and centrifuged. After they were filtering through a 0.22-µm filter, the obtained supernatants were injected into an automatic amino-acid analyzer (S433 model, Sykam, GmbH, Eresing, Germany). Two replications were conducted for each measurement.

### 3.3. RNA Extraction

Total RNA was extracted from roots using the BIOZOL Total RNA Extraction Reagent (Biozol, Eching, Germany) pBIOZOL according to the manufacturer’s protocol. RNA integrity was confirmed using an Agilent 2100 Bioanalyzer.

### 3.4. cDNA Library Preparation and Illumina Sequencing

The pooled RNA described above was used for cDNA preparation. Firstly, beads with Oligo (dT) were used to isolate poly (A) mRNA. Then, a cDNA library was prepared using Illumina’s kit according to the manufacturer’s instructions. The cDNA library from tea roots was sequenced from both the 5′ and 3′ ends, using the Illumina HiSeq™ 2000 platform (eight lanes) at the Beijing Genome Institute (BGI, Shenzhen, China). The fluorescent images were processed for sequence base-calling and the quality values were calculated using the Illumina data-processing pipeline; thereafter, 100-bp paired-end reads were obtained.

### 3.5. De Novo Assembly of Transcriptome and Analysis of Illumina Sequencing Results

Before assembly, raw reads were cleaned by removing adaptor sequences, empty reads, reads containing unknown sequences “N” with a rate more than 10%, and low-quality reads containing more than 50% bases with quality score ≤5. De novo assembly of clean reads was performed using the Trinity program [54]. Briefly, clean reads were first split into small pieces to produce contigs using de Bruijn graphs. The resulting contigs were then further joined into scaffolds using the paired-end reads. Gap filling was subsequently carried out to obtain complete scaffolds using the paired-end information to retrieve the read pairs with one read well aligned to the contigs and another read located in the gap region. Subsequently, the TGICL program [55] was used to assemble these four *Camellia sinensis* root unigenes to form a single set of non-redundant unigenes.

The expression levels of assembled unigenes were calculated using the fragments per kilobase of the transcript per million fragments mapped (FPKM) method [56]. Files containing the Illumina DNA sequences and quality scores were submitted to NCBI’s Short Read Archive (accession SRP045464). All assembled unigenes, except those shorter than 200 bp, were deposited into the Transcriptome Shotgun Assembly (TSA) Sequence Database at NCBI (GeneBank: GBKQ00000000).

### 3.6. Functional Annotation and Classification

All Illumina-assembled unigenes were annotated by assigning putative gene descriptions, conserved domains, gene ontology (GO) terms, and putative metabolic pathways based on sequence similarity with previously identified genes annotated with those details. In order to assign the predicted gene descriptions, the assembled unigenes were aligned to the plant protein dataset of NR, Swiss-Prot database, Clusters of Orthologous Groups (COGs, and the Kyoto Encyclopedia of Genes and Genomes (KEGG) pathways, using a BLASTX procedure (E-value ≤ 1 × 10^−5^). The protein functions were predicted based on the annotation information of the most similar proteins in those databases.

Functional categorization was carried out using GO terms (GO; http:// www.geneontology.org) based on the best BLASTX hits from the NR database using Blast2GO software. After obtaining GO annotation of unigenes, WEGO software was used to perform GO functional classification of all annotated unigenes.

KEGG pathway annotation was performed by sequence alignment against the KEGG database using the BLASTX algorithm.

At the same time, the orientation of Illumina sequences which failed to be obtained directly from sequencing was derived from BLAST annotations. Other sequences falling beyond the BLAST ESTScan program were used to predict the “Coding DNA Sequence (CDS)” and their orientation.

To determine the number of unique annotations in the four databases (NR, Swiss-prot, COGs, and KEGG), we filtered the annotation files to determine the redundancy in Nr-ID, Swiss-prot-ID, COG-ID, and KO-ID, respectively.

### 3.7. Identification of Differentially Expressed Genes

Differentially expressed genes between control and treatment group were identified using logarithmic ratios of Fragments Per Kilobase of transcript per Million mapped reads (FPKM) values with parameters of |log2Ratio| ≥1 and false discovery rate (FDR) ≤0.001. Finally, those genes significantly differentially expressed in both two tea cultivars were determined as differentially regulated genes.

### 3.8. Isolation of *pCsAlaDC* and Sequence Analysis

According to the information from the transcriptome database of *C. sinensis*, the full-length Open Reading Frame (ORF) of target gene was isolated by PCR amplification with specific primers (forward primer, 5′–CTCCTCGGATTCTCAAACCTCAC–3′; reverse primer, 5′– CACACAAACGATAGTAGAACCAACA–3′). A total of 500 ng of cDNA was used as the template in a 50-µL PCR reaction mixture containing 2.5 U of LA Taq DNA Polymerase, 5 µL of 10 × LA Taq buffer Ⅱ (Mg^2+^, plus), 1.6 mM dNTPs, and 0.3 µM *pCsAlaDC*-specific primers. The PCR conditions were as follows: denaturation at 94 °C for 4 min, followed by 30 cycles of 94 °C for 30 s, 60 °C for 30 s, 72 °C for 2 min, and a final extension at 72 °C for 10 min. The PCR products was purified and cloned into the pMD18-T vector (Takara, Dalian, China). The recombinant plasmid with the target gene was subsequently transferred into *Escherichia coli* (strain DHα) competent cells and sequenced to confirm the full-length of the amplified ORF.

BioEdit software was used for ORF identification and translation from nucleic acids into amino acids. The tools on ExPASy website (http://www.expasy.org/resources) were used to predict protein molecular weight, isoelectric point (ProtParam), signal peptides (SignalP), transmembrane helixes (TMHMM), secondary structures, and subcellular location (PredictProtein). Homologous sequences of the putative protein CsAlaDC were obtained from Swiss-Prot database. The amino-acid sequences were used to construct a phylogenetic tree using the neighbor-joining method with MEGA 7.0 (http://www.megasoftware.net/home).

### 3.9. Heterologous Expression and Purification of the Recombinant Protein

The full-length ORF of *pCsAlaDC* gene was amplified by PCR using specific primers (forward primer, 5′–CGGGATCCATGGAAGGGACTGTGTCAGTGCTATC–3′; reverse primer, 5′–CCCAAGCTTGTTTATGAAGATCACAATCACAATTCTCAC–3′) containing cleavage sites (the underlined bases) of *BamHI* and *HindIII* in forward and reverse primers, respectively. The PCR product and pET-28b vector were digested with the restriction enzymes mentioned above. Recycled with agarose gel electrophoresis, the target gene was sub-cloned into the pET-28b vector and the recombinants were transferred into *E. coli* BL21 (DE3) (Transgen, Beijing, China). After the sequence of target gene was confirmed, the *E. coli* BL21 strain transformed with the target gene was cultured at 37 °C in the LB medium (Lysogeny Broth) containing 50 µg/mL Kanamycin to an OD_600_ of 0.4–0.6. Isopropyl-β-d-thiogalactopyranoside (IPTG) at a final concentration of 0.5 mM was added to the cell culture, which was cultured at 16 °C overnight to induce the expression of recombinant His_6_-CsAlaDC protein. The recombinant protein was purified by a column of Ni-NTA Sepharose (Qiagen, Hilden, Germany) and detected by SDS-PAGE gel analysis.

### 3.10. Measurement of Enzymatic Activity

The protein activity was assayed by detecting the products of substrate decarboxylation [46] in the reaction mixture using UPLC-MS. Two controls were set: one for substrate Ala or Ser, the other for target the protein. To start, 100 µL of reaction mixture containing 10 µg of recombinant enzyme and 90 µL of Buffer A (10 mM alanine, 100 mM potassium phosphate, pH 7.2, 0.1 mM pyridoxal phosphate, 5 mM l-dithiothreitol, 1 mM K_2_EDTA, 10% glycerol) for alanine decarboxylation, and another 100 µL of reaction mixture containing 10 µg of recombinant enzyme and 90 µL of Buffer S (10 mM serine, 100 mM potassium phosphate, pH 7.2, 0.1 mM pyridoxal phosphate, 5 mM l-dithiothreitol, 1 mM K_2_EDTA, 10% glycerol) for serine decarboxylation were prepared and incubated at 35 °C. Then, 45 µL of the reaction mixture was taken out after 2 h and 24 h of incubation, and the reaction was stopped with 5 µL of 10% trichloroacetic acid. The mixture was diluted with 400 µL of ddH_2_O, filtrated with a 0.22-µm Millipore membrane, and analyzed using UPLC-MS after derivatization with AccQ-Tag Kit (Waters, Shanghai, China). One-way analysis of variance (ANOVA) and multiple comparisons with Latin square design (LSD) were conducted to test the differences at different time points between groups using SPSS 16.0 software. A *p*-value ≤0.01 was considered to be statistically significant.

### 3.11. Relative Quantification by Real-Time PCR

Gene expression analysis was performed with the 7500 Real-Time PCR System (Applied Biosystems, Waltham, MA, USA) with the SYBR Premix Ex Taq Ⅱ according to the manufacturer’s instruction. Primers (forward primer, 5′–GATTGGTTTGCCCGTCTATG–3′; reverse primer, 5′–ATTATGGCTGGTTTGTCCTT–3′) used for real-time PCR were designed using online software Primer-BLAST. Consequently, 20 µL of the reaction mixture contained 10 µL of SYBR Premix Ex Taq, 0.4 µL of Dye Ⅱ, 2 µL of template cDNA (40 ng/µL), and 0.4 µL of primers (10 mM). The PCR conditions were as follows: denaturation at 95 °C for 30 s, followed by 40 cycles of 95 °C for 5 s and 60 °C for 34 s, and finally, 95 °C for 15 s, 60 °C for 1 min, and 95 °C for 15 s. Relative expression of this target gene was detected with at least three independent cDNAs and analyzed using the 2^−ΔΔ Ct^ method [57] with *GAPDH* as a reference.

## 4. Conclusions

In this study, a novel gene coding for CsAlaDC was identified from the roots of tea plants by analyzing amino-acid contents and the differentially expressed genes in roots between the nitrogen starvation and control groups. The enzymatic activity of CsAlaDC protein was measured in vitro, and the results illustrated that the target protein could catalyze the decarboxylation of Ala despite of its high similarity with SDC from other species. The transcription levels of this novel *CsAlaDC* gene in roots were significantly higher than those in leaves. That may explain why theanine biosynthesis preferentially occurs in the roots of tea plants. Furthermore, the expression of the gene was upregulated when nitrogen was present, suggesting that theanine biosynthesis was regulated by nitrogen supply and closely related to nitrogen metabolism of *C. sinensis*. The results of this study provide important supplements to the theanine biosynthesis pathway and might be helpful for a better understanding of the differences in theanine accumulation between *C. sinensis* and other species.

## Figures and Tables

**Table 1:** Summary of messenger RNA (mRNA) detected under nitrogen starvation conditions.

	Zhongcha 302	Zhongcha 108
Unique gene detected	91,650	98,406
Significantly changed genes	7985	17,824
Upregulated	3288	15,785
Downregulated	4697	2039
Shared genes (upregulated)	742	
Shared genes (downregulated)	128	

## Supplementary Materials

Supplementary Figure S1: Multiple alignment of the protein sequence of pCsAlaDC with the characterized SDCs from other species.
