# Supplementary material for: Identification of a Novel Gene Encoding the Specialized Alanine Decarboxylase in Tea (Camellia sinensis) Plants
Source: Molecules. 2019 Feb 1;24(3):540. doi: 10.3390/molecules24030540 (PMC6384637; doi:10.3390/molecules24030540)
Supplement: Supplementary file 1 [file molecules-24-00540-s001.pdf]

molecules

Supplementary Material

Identification of a Novel Gene Encoding the Specialized Alanine Decarboxylase in Tea (*Camellia sinensis*) Plants

Peixian Bai [1,2,†], Kang Wei [1,†], Liyuan Wang [1,*], Fen Zhang [1], Li Ruan [1], Hailin Li [1], Liyun Wu [1] and Hao Cheng [1,*]

[1] National Center for Tea Improvement, Tea Research Institute Chinese Academy of Agricultural Sciences, Hangzhou 310008, China; baipeixian2018@outlook.com (P.B.); weikang@tricaas.com (K.W.); zhangfen2008.cool@163.com (F.Z.); ruanli@tricaas.com (L.R.); lihailin@tricaas.com (H.L.); wuly@tricaas.com (L.W.); chenghao@tricaas.com (H.C.)

[2] College of Horticulture and Forestry Science, Huazhong Agricultural University, Wuhan 430070, China;

* Correspondence: chenghao@tricaas.com (H.C.); wangly@tricaas.com (L.W.); Tel.: +86-571-866-53-169 (H.C.); +86-571-866-50-575 (L.W.)

† These authors have contributed equally to this work.

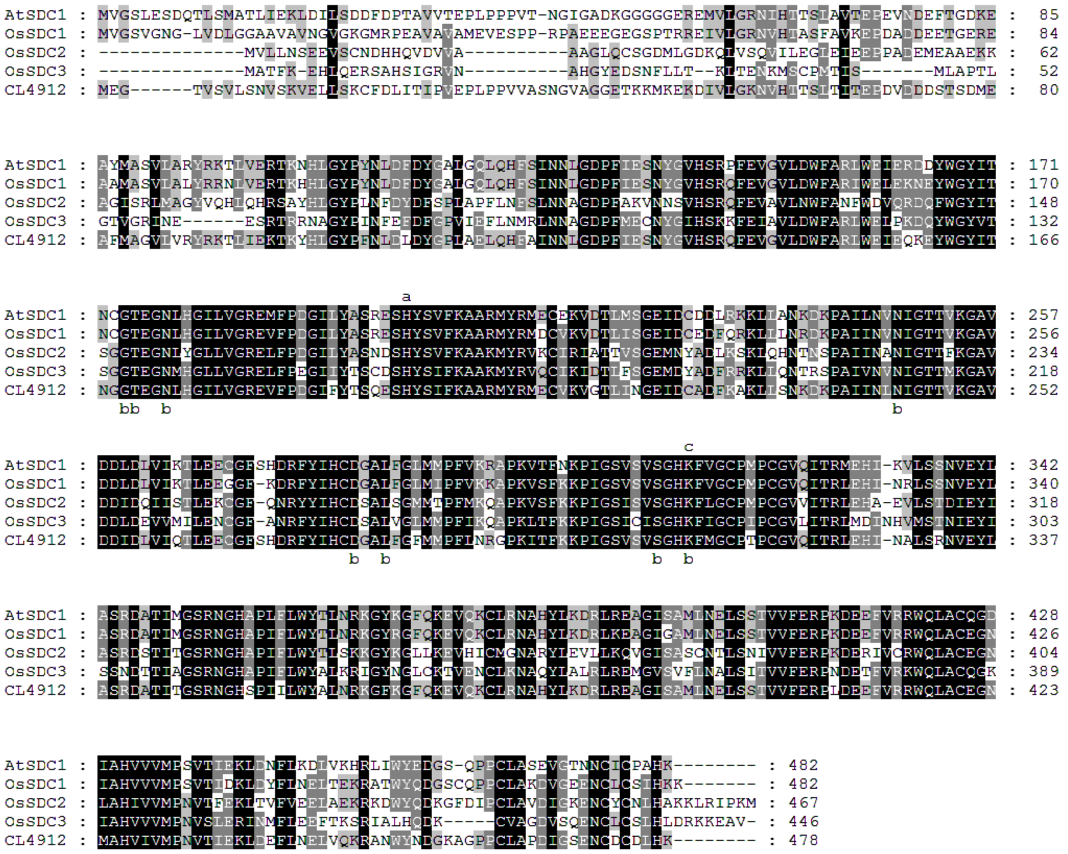

Supplementary Figure S1. Multiple alignment of the protein sequence of pCsAlaDC with the characterized SDCs from other species. Identical amino acids are highlighted in black, and similar amino acids are shaded in gray. The conserved substrate binding site, pyridoxal 5′-phosphate binding pocket, and catalytic residue is marked with a, b and c, respectively. The UniProt knowledgebase accession number are as follows: AtSDC1(sp|Q9MA74); OsSDC1(sp|Q6ESZ9); OsSDC2(sp|Q8RV06); OsSDC3(sp|Q7X8D4). Arabidopsis thaliana, At; Oryza sativa, Os.

© 2019 by the authors. Submitted for possible open access publication under the terms and conditions of the Creative Commons Attribution (CC BY) license (http://creativecommons.org/licenses/by/4.0/).
